# Quantifying the Improvement of Surrogate Indices of Hepatic Insulin Resistance Using Complex Measurement Techniques

**DOI:** 10.1371/journal.pone.0039029

**Published:** 2012-06-22

**Authors:** John G. Hattersley, Matthias Möhlig, Michael Roden, Ayman M. Arafat, Christian v. Loeffelholz, Peter Nowotny, Jürgen Machann, Johannes Hierholzer, Martin Osterhoff, Michael Khan, Andreas F. H. Pfeiffer, Martin O. Weickert

**Affiliations:** 1 Warwickshire Institute for the Study of Diabetes, Endocrinology and Metabolism, University Hospitals Coventry and Warwickshire NHS Trust, Coventry, United Kingdom; 2 Human Metabolic Research Unit, University Hospitals Coventry and Warwickshire NHS Trust, Coventry, United Kingdom; 3 Department of Clinical Nutrition, German Institute of Human Nutrition, Potsdam-Rehbruecke, Germany; 4 Department of Endocrinology, Diabetes and Nutrition, Charité-University-Medicine Berlin, Germany; 5 Institute for Clinical Diabetology, German Diabetes Center (Leibniz Center for Diabetes Research), and Department of Metabolic Diseases, University Clinics and Heinrich-Heine University Düsseldorf, Düsseldorf, Germany; 6 Section on Experimental Radiology, Department of Diagnostic and Interventional Radiology, Eberhard-Karls University Tübingen, Tübingen, Germany; 7 Diagnostic and Interventional Radiology, Klinikum Ernst von Bergmann, Academic Teaching Hospital, Charité University Medicine Berlin, Potsdam, Germany; 8 Clinical Sciences Research Institute, Warwick Medical School, University of Warwick, Coventry, United Kingdom; Universita Magna-Graecia di Catanzaro, Italy

## Abstract

We evaluated the ability of simple and complex surrogate-indices to identify individuals from an overweight/obese cohort with hepatic insulin-resistance (HEP-IR). Five indices, one previously defined and four newly generated through step-wise linear regression, were created against a single-cohort sample of 77 extensively characterised participants with the metabolic syndrome (age 55.6±1.0 years, BMI 31.5±0.4 kg/m^2^; 30 males). HEP-IR was defined by measuring endogenous-glucose-production (EGP) with [6–6^2^H_2_] glucose during fasting and euglycemic-hyperinsulinemic clamps and expressed as EGP*fasting plasma insulin. Complex measures were incorporated into the model, including various non-standard biomarkers and the measurement of body-fat distribution and liver-fat, to further improve the predictive capability of the index. Validation was performed against a data set of the same subjects after an isoenergetic dietary intervention (4 arms, diets varying in protein and fiber content versus control). All five indices produced comparable prediction of HEP-IR, explaining 39–56% of the variance, depending on regression variable combination. The validation of the regression equations showed little variation between the different proposed indices (*r* ^2^ = 27–32%) on a matched dataset. New complex indices encompassing advanced measurement techniques offered an improved correlation (*r* = 0.75, *P*<0.001). However, when validated against the alternative dataset all indices performed comparably with the standard homeostasis model assessment for insulin resistance (HOMA-IR) (*r* = 0.54, *P*<0.001). Thus, simple estimates of HEP-IR performed comparable to more complex indices and could be an efficient and cost effective approach in large epidemiological investigations.

## Introduction

Hepatic insulin resistance (HEP-IR) is emerging as a central determinant of whole-body insulin resistance, fatty liver disease, dyslipidemia and Type 2 Diabetes [1]–[3] and is being suggested as a key treatment target in new drug development for diabetes and its precursor states. Therefore, accurate means of assessing and quantifying the degree of HEP-IR are urgently required.

The gold standard method for measuring HEP-IR is the performance of tracer dilution studies incorporating stepped clamps, which is not feasible in larger metabolic studies and would not be suitable for routine clinical use [4]. Considerable efforts have been devoted to identifying simpler and less resource intensive means of estimating HEP-IR. Estimating HEP-IR as the product of fasting endogenous glucose production (EGP), as measured using tracer dilution technique, and fasting plasma insulin (FPI) levels has been proposed and used as the next best method for measuring HEP-IR [2], [5]. Unfortunately, this method still requires highly skilled staff with specialised equipment that is not available to many clinical centres. In addition, stable isotope methods are typically time consuming and expensive, thus making them inapplicable for use in epidemiological and/or larger metabolic studies. Therefore, the development and validation of simple and cost effective indices for estimating HEP-IR that identifies individuals with a high probability of hepatic insulin resistance is clinically relevant.

A large multi-centre European consortium [2] has recently proposed a novel index for the estimation of HEP-IR, using EGP multiplied by FPI as the measure of HEP-IR against which the index was compared. This index was derived using linear regression techniques [6], resulting in a model which combined simple phenotype information (body mass index (BMI) and estimated fat-mass) with plasma measurements (insulin and high density lipoprotein (HDL) cholesterol). The index correlated reasonably well with the data sets in the original study, between *r* = 0.53 to 0.65 (*P*<0.001), depending on data set partitioning. Using results from an alternative dataset of overweight and obese non diabetic subjects with the metabolic syndrome [7], as defined by International Diabetes Federation criteria [8], we aimed to validate both the proposed estimation index [2] as well as several potential alternatives proposed in this study by comparing performance against an accepted direct HEP-IR measurement using tracer dilution.

Critical factors when developing new indices include: (i) the ability of the model to accommodate the original data, often referred to as the goodness-of-fit; and (ii) the ability to accurately predict the response of new patients in the face of potentially large variation in signals due to inherent inter and intra-patient variability and measurement error [9]. Therefore, validation of any index against other data sets and/or using statistical approximations (e.g. cross-validation, boot strapping) is essential [4]. The subjects in this study underwent a dietary intervention after initial baseline measurements. Since it is not appropriate to assume that the intervention had no effect on HEP-IR the cohorts were not pooled into a single dataset to achieve a greater sample size. Therefore, in this study the measurements obtained have been maintained separately, creating two datasets which contained the same subjects with variable measurements taken before and after an isoenergetic dietary intervention varying in dietary protein and fiber content [7]. This data partition allows the regression to be validated and to investigate the robustness and insensitivity of the proposed indices to real life conditions; further, if an index were unable to maintain accuracy given such a relatively mild intervention, it is unlikely to perform in completely independent cohorts where, apart from the diets, various other factors are likely to influence the results.

## Methods

### Study Population

Details of the here investigated study population have been published [7], [10] and the trial was registered at clinicaltrials.gov as NCT00579657. The Ethics Committee of the University of Potsdam approved the study (BMBF FKZ 0313826). All investigations were preformed in agreement with the declaration of Helsinki. All participants had given written informed consent. The baseline characteristics of the subjects with a full data set for the measurement of whole-body insulin sensitivity (using euglycemic-hyperinsulinemic clamps), hepatic insulin resistance (HEP-IR; using stable isotope methods) and further parameters are presented in Table 1. All participants were characterized using oral glucose tolerance tests (oGTT) prior to the study (normal glucose metabolism (NGM), *n* = 38; impaired fasting glucose (IFG), *n* = 24; impaired glucose tolerance (IGT), *n* = 3; IFG+IGT, *n* = 12; diabetes, *n* = 0). All participants were overweight with a body-mass-index (BMI) ≥25 kg/m^2^ and fulfilled the criteria of the metabolic syndrome according to IDF criteria [8], and 53 of the participants were obese (BMI ≥30 kg/m^2^).

**Table 1:** Characteristics of the study population cohort for regression.

	Baseline	Validation	*P* value
Number of subjects (n)	77	74	
Sex (males/females)	30/47	30/44	
Age (years)	55.6±1.0	57.0±1.0	0.33
Weight (kg)	89.7±1.8	87.7±1.6	0.39
BMI (kg/m^2^)	31.5±0.4	30.7±0.3	0.13
Waist (cm)	101.7±1.3	99.3±1.1	0.15
Use of antihypertensive and/or lipid lowering drugs (n)	38	31	0.36
REE (kcal/day)	1495±33	1500±39	0.91
Insulin resistance			
Fasting EGP basal (mg·kg^−1^·min^−1^)	1.65±0.02	1.68±0.02	0.25
Clamp suppression EGP (mg·kg^−1^·min^−1^)	0.27±0.03	0.34±0.03	0.08
EGP * FPI (mg·kg^−1^·min^−1^)* (mU/L)	15.7±0.9	17.7±1.0	0.14
MCR (mL·kg^−1^·min^−1^)	4.88±0.24	4.4±0.2	0.11
M-value (mg·kg^−1^·min^−1^)	4.2±0.2	3.9±0.2	0.21
Fasting plasma glucose (mg/dl)	86.1±0.9	84.2±0.8	0.10
Fasting plasma insulin (mU/L)	9.7±0.6	10.6±0.6	0.30
Body composition			
VAT (L)	4.5±0.2	4.1±0.2	0.34
NVAT (L)	16.3±0.6	14.1±0.4	0.003
Intrahepatic fat content (%)	8.2±1.1	6.6±0.8	0.26
Total body fat mass (kg)	36.3±1.1	33.1±0.8	0.023
Lean mass (kg)	53.4±1.4	54.5±1.4	0.057
Biomarkers			
ASAT (U/L)	21.7±0.7	20.3±0.6	0.12
ALAT (U/L)	23.7±1.7	23.5±2.1	0.93
GGT (U/L)	23.5±2.0	23.3±2.1	0.93
CK-18 (U/L)	183.7±12.5	159.4±9.7	0.13
DHEA-S (ng/mL)	1078±85	1079±58	1.0
Total cholesterol (mmol/L)	5.3±0.1	5.2±0.1	0.67
HDL (mmol/L)	1.3±0.0	1.2±0.0	0.044
LDL (mmol/L)	3.4±0.1	3.4±0.1	0.50
Triacylglycerols (mmol/L)	1.1±0.1	1.4±0.1	0.014
FFA (mmol/L)	0.7±0.0	0.7±0.0	0.91
Adiponectin (µg/mL)	12.2±0.7	15.4±1.2	0.022
Leptin (ng/mL)	18.2±1.4	14.5±1.1	0.043

BMI, body mass index; REE, resting energy expenditure; EGP, endogenous glucose production; FPI, fasting plasma insulin; MCR, metabolic clearance rate of glucose; VAT, visceral adipose tissue; NVAT, non-visceral abdominal adipose tissue; ASAT, aspartate amino transferase; ALAT, alanine amino transferase; GGT, gamma-glutamyl transferase; CK-18, cytokeratin 18; DHEA-S dehydroepiandosterone sulphate; HDL; high density lipoprotein; LDL, low density lipoprotein; FFA, free fatty acids.

n = 77 overweight and obese non-diabetic participants with metabolic syndrome. Validation analyses were performed in a semi-independent cohort, investigating the same participants that participated in an isoenergetic dietary intervention [8], 6–18 weeks after the baseline measurements (n = 74). Analyses were performed using one-way ANOVA.

Longitudinal data on the same subjects measured after 6–18 weeks were used for validation of the here proposed indices. Participants were subjected to an 18 weeks isoenergetic dietary intervention varying in dietary fibre and protein contents, and comparable fat contents (30% of energy intake). Subjects were group matched according to age, gender, waist circumference, body mass index (BMI), and drug intake, and assigned to either a control group [percent of energy intake, protein (P) 17%, carbohydrates (C) 51–52%, dietary fibre (F) 14–15 g]; a high-cereal fiber group (P 17%, C 51–52%, F 41–43 g); a high-protein group (P 26–28%, C 43–45%, F 13–14 g); or a diet moderately high in both protein and dietary fiber (P 22–23%, C 44–46%, F = 26 g) [7].

### Euglycemic Hyperinsulinemic Clamps for the Measurement of Whole-body Insulin Sensitivity

Participants arrived at the metabolic unit between 07∶15 and 08∶30 am after a 10-h overnight fast. No intake of food or any drinks apart from tap water was allowed within the last 12 h before the studies. Two intravenous catheters were inserted into contralateral forearm veins. The arm at which blood samples were drawn was placed into a warming box (65°C) throughout the clamp studies. After administration of an insulin bolus at –10 min (individually adjusted according to the body surface area of the participants), euglycemic-hyperinsulinemic clamps were performed at a constant insulin infusion rate of 40 mU·m^–2^·min^–1^, for at least 120 min until steady state conditions were achieved. Steady state was defined as stable glucose infusion rates (GIR) over at least 30 min, together with stable plasma glucose concentrations (range of 4.4±0.4 mmol/liter). Whole-body glucose disposal was calculated from the glucose infusion rate and was expressed as insulin-mediated glucose uptake (M-value). Blood samples were drawn at timed intervals during the clamps, immediately chilled, centrifuged, and the supernatants were stored at –80°C until analysis.

### Stable Isotope Studies for the Measurement of Hepatic Insulin Resistance

For the measurement of hepatic endogenous glucose production (EGP; given in mg·kg^–1^·min^–1^), a primed [0.06 (mg)×body wt (kg)×fasting plasma glucose (mg/dl), from –120 to –115 min], continuous [0.27 (mg)×body wt (kg), from –115 to +320 min] infusion of [6,6-^2^H_2_]glucose 99% (Euriso-Top, Saarbrücken, Germany) was administered. A basal period of 100 min was allowed for tracer equilibration, as described [11]. The priming dose was adjusted to fasting glucose concentrations to avoid overestimation of glucose production rates. Rates of EGP were determined from the tracer infusion rate of D-[6,6-^2^H_2_]glucose and its enrichment to the hydrogen bound to carbon 6 divided by the mean percent enrichment of plasma D-[6,6-^2^H_2_]glucose. Because both GIRs and plasma glucose levels were held constant during the steady state phase of the clamps, steady-state equations were appropriate for the calculation of EGP [11]. HEP-IR was then calculated as the product of fasting EGP and fasting plasma insulin [5]. Further details have been published [7], [11], [12]. Euglycemic hyperinsulinemic clamp conditions resulted in significant and near complete suppression of EGP in all subjects (1.64±0.02 mg·kg^–1^·min^–1^ (baseline) vs 0.26±0.03 mg·kg^–1^·min^–1^ (steady state), *P*<0.00001), as could be expected in non diabetic participants.

### Measurement of Body Composition and Liver Fat Content

Magnetic resonance examinations were performed on a 1.5 T whole body imager (Magnetom Avanto, Siemens Healthcare, Erlangen, Germany) as described [7], [13]. For quantification of abdominal adipose tissue, an axial T1-weighted fast spin echo technique with an echo train length of 7 was applied. Measurement parameters were: echo time (TE) = 12 ms, repetition time (TR) = 490 ms, slice thickness 10 mm, 5 slices per sequence, 10 mm gap between the slices. A 256×178 matrix was recorded in a measuring time of 12 s and images were recorded from the femoral head to the head of the humerus (between 26 and 30 slices, depending on the size of the volunteer). Volunteers were in prone position with the arms extended. Post-processing was performed by a semiautomatic segmentation program (Matlab 6.5) by determination of noise, lean tissue and adipose tissue. Visceral adipose tissue (VAT) was determined by manually drawing a region of interest in the original image, and non visceral abdominal adipose tissue (NVAT) was calculated as difference between total abdominal adipose tissue and VAT, thus including adipose tissue around the heart and intermuscular adipose tissue.

Proton magnetic resonance spectroscopy (^1^H-MRS) for the measurement of hepatic lipid content was performed as described [7]. In brief, lipid content in the liver was measured by localized ^1^H-MRS from a volume of interest (VOI) within the posterior part of segment 7 of the liver. A single segment of the spine array coil was used for acquisition of the spectroscopic data. For volume selection, a single voxel STEAM technique was applied. Measurement parameters were TR = 4 s, TE = 10 ms, TM = 15 ms, VOI 3.0×3.0×2.0 cm^3^. Thirty-two acquisitions were recorded to obtain a sufficient SNR in a measuring time of 2∶08 min. In order to minimize line broadening due to breathing, volunteers were requested to breathe within the TR interval and to be in expiration during each data acquisition. Shimming of the VOI was performed in the automatic mode and the volunteers were requested to breathe flatly. Signal integrals of water (H_2_O at 4.8 ppm) and lipids (CH_2_ and CH_3_ at 1.25 ppm and 0.95 ppm) were quantified manually in fixed frequency intervals (water: 3.1–6.2 ppm, lipids: 0.5–1.8 ppm). HL_spec_ values were calculated by the ratio Int(lipids) over Int(lipids+water).

Total body fat and lean mass were measured by using air-displacement plethysmography (BOD POD, Cosmed, Rome, Italy), as described [7].

### Biomarkers in Blood

Routine laboratory markers were measured using standard methods in the research laboratories of the German Institute of Human Nutrition. Glucose concentrations were measured in venous blood (ABX Pentra 400, ABX Diagnostics, Montpellier, France), and additionally, for the performance of clamp studies, in arterialized blood samples. Arterialized plasma glucose concentrations were measured immediately, using the glucose oxidase method (Dr. Müller Super-GL glucose analyzer, Freital, Germany). Adiponectin (ADI) concentrations were measured by enzyme-linked immunosorbent assay (Biovendor, Nashville, TN) [intra-assay coefficient of variation (CV) 6.7%]. Free fatty acids, cholesterol, LDL and HDL cholesterol, and triglycerides were analysed using Cobas Mira (Roche, Lörrach, Germany); intra-assay CV: free fatty acids, 10.5%; cholesterol, 5.1%; HDL cholesterol, 5.4%; and triglycerides, 5.1%). Cytokeratin 18 (CK-18), commonly considered as a marker of cell death, has been recently proposed as independent predictor of non-alcoholic steatosis hepatis (NASH) [14]–[16] and was therefore included in the analyses. CK-18 was measured using M30-Apoptosense ELISA (Peviva, Bromma, Sweden; intra-assay and inter-assay CV <10%). Dehydroepiandrosterone sulphate (DHEA-S) was measured using a radioimmunoassay (DSL-2700 DHEA-S-7 RIA, Oxon, UK; intra-assay CV 3%, inter-assay CV 5%).

### Statistical Methods

The statistical methods centre on the use of linear regression to identify potential indices of HEP-IR. The dataset used [7] contains over 200 potential predictor variables for the regression models. In order to address the issue of practicality and cost the predictors variables are separated into subsets on the basis of cost and the inherent difficulty in measuring the variable in question, with variables that may be measured through standard anthropometric techniques or directly from blood samples being referred to as simple variables, and more cost intensives variables requiring specific expertise and laboratory equipment being referred to as complex variables. The specific variables used are listed in the relevant parts of the results section. As in previous studies [2] forward step-wise linear regression was performed on each variable set using EGP multiplied by fasting plasma insulin (EGP*FPI) as the outcome variable in each model. Variables were rejected if additional variable contribution could be ascribed to chance with *P*>0.05. In order to avoid potential bias during the regression analyses all variables were blinded to the scientist performing the analysis. Variables were checked for normality using Shapiro-Wilk test and natural logarithmic transformation was used if required, or to maintain consistency with other authors. The index EGP*FPI was not normally distributed and was therefore log transformed, which is also in accordance with [2]. Collinearity of variables was determined using variance inflation factor (VIF). For the regression all data that had a Cook’s distance of greater than 0.5 were considered outliers and removed from the dataset to perform stability analysis. Stability of the regression variables was determined using a bootstrap process (250 iterations). In order to ensure that the indices are not unduly influenced by patient’s drugs treatment the validation group was separated into two cohorts (drugs, or no drugs). By definition, each sub-group was independent; therefore the Fisher Z transforms of the respective correlations were found and a 95% confidence level (α = 0.05) was used to decide whether the difference in correlation was statistically significant.

Baseline characteristics between the regression and the validation cohort were compared using one-way analysis of variance (ANOVA). The total area under the curve (AUC) was calculated using the trapezoidal method. Statistical analyses were performed by using SPSS version 19 (SPSS Inc, Chicago, USA).

### Validation

In order to compare each index with the regression data set Pearson correlations (r) and the related co-efficients of determination (*r^2^*) were calculated for regression and validation. In addition, the r^2^ adjusted for the number of variables incorporated (adj- *r^2^*) was calculated for each regression index, allowing comparison of indices with respect to the accuracy fit and parameter numbers.

For validation two additional statistics have been included to assess the predictive accuracy: the root mean square of the error (RMSe, also referred to as the standard estimate of the error) and the co-efficient of variation of the RMSe. Defined as
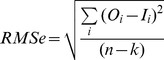
where O_i_ and I_i_ are the i^th^ subject of the outcome variable, log(EGP*FPI) in this case, and index estimate, *n* is the number of subjects in the validation dataset and *k* the number of parameters estimated. The RMSe is an indication of the variability of data points with respect to the regression line, which has the benefit of being in the same units as the original outcome variable (i.e. log(FPI*EGP)) allowing direct comparison between indices [17]. It can be considered indicative of a typical error in the estimated value, thus allowing models to be compared. The Coefficient of Variation of the RMS error is also included



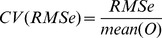
where *O* is the outcome variable as defined above. This can be interpreted as a percentage average error. All subjects in the validation data set did not include all measurements required for each regression equation; therefore the validation set was restricted to those individuals that had measurements for all the variables required for all indices.

## Results

**Figure 1:**
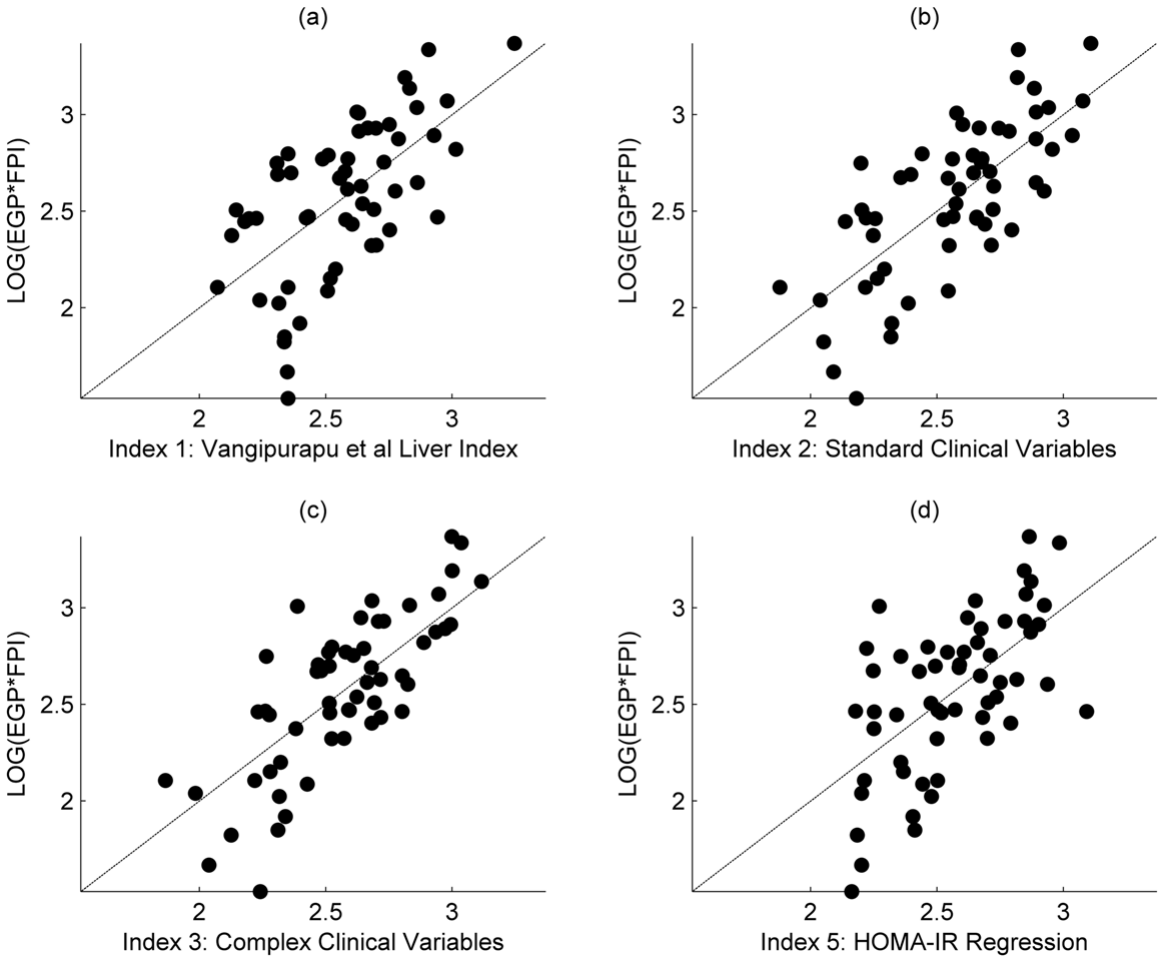
Comparison of regression against outcome variable. a) Hepatic insulin resistance (HIR) Index as described in Vangipurapu et al.; b) HIR Index generated from simple clinical measurements; c) HIR index from the regression on the complex measurement set. d) HOMA-IR Regression index.

**Figure 2:**
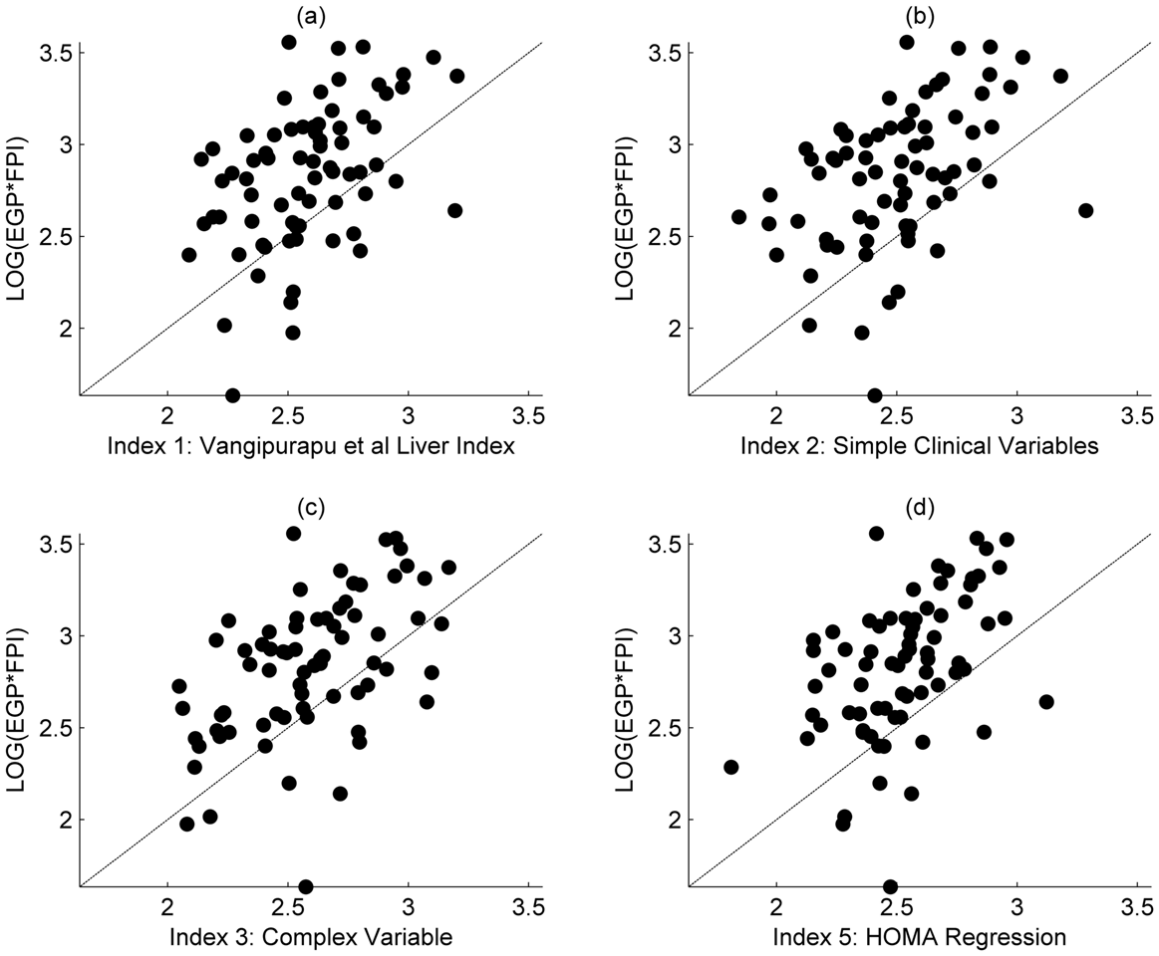
Comparison of regression against outcome variable, validation only. a) Hepatic insulin resistance (HIR) Index as described in Vangipurapu et al.; b) HIR Index generated from simple clinical measurements; c) HIR index from the regression on the complex measurement set. d) HOMA-IR Regression index.

Baseline characteristics of the investigated participants are presented in Table 1. Five indices for the estimation of hepatic insulin resistance have been indentified using step-wise linear regression, as described in the statistical analysis section. The first is the direct application of a recently proposed index of Vangipurapu et al. [2], and the remaining four are derived for this paper. The details of each model and the variables included in the regression analyses are provided below. Table 2 provides a summary of the regression statistics and results from the validation process. Figure 1 shows the comparison of the respective regressions against the outcome variable, EGP multiplied by FPI. Results from the validation data set are shown in Figure 2.

**Table 2:** Output statistics for the regression (n = 77) and when the indices are applied to the design and validation dataset (n = 74).

Index	Description	Regression			Validation			
		r^a^	r^2^	adj. r^2^	r^a^	r^2^	RMSe	CV%
1	Vangipurapu et al. index (eq. 1)	0.62	0.39	0.34	0.52	0.27	0.48	17.0
3	Standard clinical variables (eq. 2)	0.73	0.54	0.51	0.55	0.30	0.51	18.2
5	Extensive clinical variables (eq. 3)	0.75	0.56	0.54	0.56	0.32	0.46	16.3
4	HOMA-IR	0.58	0.33	N/A	0.54	0.30	N/A	N/A
5	HOMA-IR Regression (eq. 4)	0.62	0.39	0.38	0.54	0.30	0.49	17.6

r is Pearson’s correlation co-efficient, r^2^ is the co-efficient of determination, adj- r^2^ is the adjusted r^2^, RMSe the root mean squared of the error and CV the co-efficient of variation of the RMSe; the mean(std. dev) of outcome variable (log EGP*FPI) for the validation data set 2.83 (0.46). ^a^statistically significant at below P<0.001.

### Index 1: Vangipurapu et al. Liver Index

The Vangipurapu et al. Liver Index [2] can be described by the following equation:
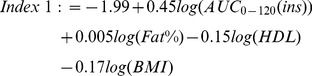
(1)where AUC_0–120_(ins) is the insulin total Area-Under-Curve between 0 and 120 min, (fat%) is the percent mass of total body fat, and HDL is the high density lipoprotein (HDL) cholesterol measurement. Although additional insulin measurements were available in our study, to be consistent with the original manuscript AUC was calculated from three measurements at time points 0, 30 and 120 minutes only. To avoid potential inconsistency in methodology and allow comparison between the datasets the Vangipurapu et al. model was applied to our dataset using a linear regression fit, without stepwise removal or inclusion. If this were not done applying the Vangipurapu et al. model would result in artificially reduced correlations with the design data set, due to the natural variation between the original Vangipurapu et al. data and the design data set of this study. With reference to Table 2, regression eq. 1 resulted in a medium strength correlation (*r*) of 0.62 (*P*<0.001) within an adjusted-*r^2^* of 0.34 when applied to the regression dataset. This decreased to 0.52 (*P*<0.001) when the index (eq. 1) was applied to the validation data set and an RMSe of 0.48, which is approximately 17% of the mean value. Index 1 (eq. 1) when populated with the original Vangipurapu et al. constants [2] achieved an correlation of r = 0.53, *P*<0.001 for both datasets, which is marginally lower as compared with the original Vangipurapu et al paper, but consistent with the analysis above.

### Index 2: Standard Clinical Measurements (Simple Variable)

In this studies dataset [7] several additional variables were measured that were not available to previous studies, and were thus added to the candidate predictors for the step-wise regression. The candidate predictors were added in a two phase approach. Set one was indicative of simple or cheap measurements, the second set included more expensive measurements or ones that required specialised training or equipment to perform. For Index 2 only the simple predictors were considered which included the Vangipurapu et al. variables as specified in Index 1, with the addition of adiponectin (ADI), fasting oGTT insulin and glucose levels, age, waist circumference, sex and whether the subject was IGT and/or IFG
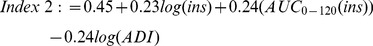
(2)where Ins_0_ is the fasting insulin level as obtained on oGTT study days, AUC_0–120_(ins) is the insulin total Area-Under-Curve between 0 and 120 min as defined above, and ADI is serum adiponectin. The variance inflation factor of Ins_0_ and AUC_0–120_(Ins) was tested to ensure that there was no collinearity, and in both cases the variance influence factor (VIF) in the above index was less than 2. In terms of the regression statistics (Table 2) this index improved on both the Vangipurapu et al index (*r* = 0.73, adj-*r^2^* = 0.51, *P*<0.001) during the regression to the design dataset; however, this is not reflected in the correlation analysis (*r* = 0.55, *P*<0.001) nor CV-RMSe 18.3% when performed against the validation data.

### Index 3: Extensive Clinical and Biochemical (Complex Variable)

For the previous two above proposed indices a simple variable set was chosen with a consideration on cost and the availability of gold standard measurement techniques; in contrast, irrespective of methodological considerations for Index 3 more complicated variables were included that were thought to have a strong influence on HEP-IR. The variables included are those defined for the Vangipurapu et al. Index 1 with the addition of intrahepatic fat content (IHL), visceral and non-visceral adipose fat mass, total fat and lean mass, biomarkers such as CK-18 and DHEA-S [18], [19], and whole-body insulin sensitivity expressed as M-Value. This variable combination provided the following index
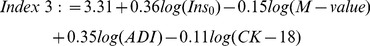
(3)with variables as defined above. This index resulted in the highest correlation during the stepwise regression (*r* = 0.75, adj-r^2^ = 0.54, *P*<0.001) and the highest correlation to the validation dataset (*r* = 0.56, *P*<0.001). When compared to the outcome variable, log(EGP*FPI), mean resulted in the lowest co-efficient of variation of 16%.

It is surprising that whilst some variables, notably IHL *r* = 0.39 (*P*<0.001) and VAT *r* = 0.36 (*P*<0.001), showed some correlation with the outcome measure (EGP*FPI), they were not supported as being significant during the regression analysis.

### Index 4 and 5: Homeostasis Model Assessment for Insulin Resistance (HOMA-IR)

A standard index for insulin resistance is the well documented HOMA-IR index [20], which simply uses FPI and fasting plasma glucose (FPG) measurements to determine whole-body insulin resistance. To allow independent comparison HOMA-IR was correlated directly against the design and validation datasets (referred to as Index 4) and a simple linear regression using the HOMA-IR was developed to evaluate the benefits of rescaling HOMA-IR through a simple transform to values within the range of the HEP-IR estimates; resulting in the following affine relationship:
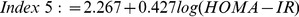
(4)Unsurprisingly, HOMA-IR directly applied to the design data showed the lowest correlation (*r* = 0.58, *P*<0.001), whilst when the additional regression parameters were used, this improved marginally (*r* = 0.62, *P*<0.001). Interestingly, when used against the validation dataset the HOMA-IR index showed a correlation (*r* = 0.54, *P*<0.001, CV-RMSe = 17.6%) higher than that of the Vangipurapu et al Index and comparable with the new indices developed in this paper using more extensive clinical measurements.

In addition to the previously described indices an index developed by Abdul-Ghani et al. [21] which incorporates the area under the curve of plasma insulin and glucose, as measured during the first 30 minutes of an oGGT, was analysed. The index produced low correlation with our regression dataset (*r* = 0.28, *P*<0.001), which is believed to be due to differences in the investigated cohorts; in our cohort of strictly overweight and obese subjects with the metabolic syndrome, a small but statistically significant difference was detected between fasting plasma insulin as measured on the study days when stable isotope experiments/clamps were performed as opposed to oGGT fasting insulin that was measured after an overnight fast following a carbohydrate challenge for 2 days (9.7±0.6 mU/L vs. 10.7±0.6, *P* = 0.044). This is likely to enforce dynamics that may offer insight into the low correlation of the Abdul-Ghani et al. index in our cohort.

As can be seen in Table 1, a relevant number of the subjects in both the validation and regression datasets where on drugs for either lipid reduction or hypertension, which may have an impact on liver function. To ensure the subjects drug regime did not impact the predictive quality of the regression, the regression dataset was split into two subgroups (drug and non-drug) and each index was applied separately to the subgroups. Using the Fisher Z Transform none of correlations where found to be significantly different at a 95% confidence level (α = 0.05). However, it must be noted that in each case the subgroups were diminished in size (drug subgroup, n = 36; no-drugs subgroup, n = 38).

## Discussion

A new index for the prediction of HEP-IR has been proposed recently [2]; however, validation in independent cohorts is necessary to ensure clinical applicability [2], [4]. In the present study we show that the recently proposed index of Vangipurapu et al. reasonably predicted HEP-IR in our cohort of well characterized overweight and obese participants with the metabolic syndrome. We show comparable results using the Vangipurapu et al. index and improved goodness-of-fit using several new indices and variable combinations. When trying to further improve the index by using parameters such as liver fat content, body fat distribution, and several biomarkers related to hepatic insulin resistance [18], [19] the predictive value of the index further improved (*r^2^* = 56%) but the additional gain of accuracy is obtained at considerable increased costs due to the state of the art methodology needed. Furthermore, when validating the improved index in our semi-independent cohort, the complex measures did not yield a relevant increase in predictive capability (*r^2^* = 32%), compared to the other presented indices. Adiponectin appeared to significantly contribute to HEP-IR predictions in several of our indices, which is consistent with the known negative correlation between adiponectin and EGP [22], and the known value of adiponectin measurements to predict insulin sensitivity in obese subjects [23]. However, additional parameters that would have been expected to show strong predictive value such as liver fat (IHL), and DHEA-S, recently proposed as a biomarker for hepatic fat content [18], [19], showed correlation with the outcome measure but, surprisingly, were not significant in the regression analyses, although there was further improvement when forced manually into the model. This however, would be expected from an increase in the number of variables and can potentially lead to over-fitting. The finding that IHL did not strongly predict HEP-IR is interesting, potentially supporting the concept of intrahepatic lipid partitioning with H_1_-spectroscopy not being able to differentiate between deleterious versus metabolically more neutral accumulation of intrahepatic fat depots [24], [25].

Results of the regression analysis are shown in Fig. 1. In each case the index describes the distribution of patient response adequately, with Index 3 appearing the most appropriate assuming a linear relationship between the outcome and predictors. This is supported by the correlation and co-efficient of determination as displayed in Table 2. However, the validation procedure (Fig. 2) demonstrates the difficulty in predicting patient response in separate cohorts, with each index showing a marked bias towards over predicting the HEP-IR outcome variables of EGP*FPI. Whilst it is expected that there will be variation when applying any index to a new dataset, due to inherent natural variation and measurement noise, it cannot be discounted that in our study the dietary intervention may have impacted the dynamics of the variables used. Otherwise, any useful index should be insensitive to simple changes in diet, given that generalizability to an independent dataset is the final aim. Although, index 3, incorporating the complex variables, could strictly be considered the strongest candidate, as the variation seen between each index when applied to the validated data set was minimal, the range of explained variation was only 5% (i.e. with reference to Table 2, r^2^ is between 0.27 and 0.32); it should not be implied from these results that any one of the proposed markers offered a clear advantage in predicting HEP-IR, and that all here investigated predictors to estimate HEP-IR are an approximation but cannot substitute the measurement of HEP-IR using state of the art methods. However, when parsimony is considered a simple linear index containing a measurement of HOMA-IR only could offer practical advantages, although care should be taken in clinical situations that have been shown to influence glucose tolerance/fasting insulin levels such as chronic renal failure [26] including its mild- to moderate stages [27]. Furthermore, if absolute values are required over relative relationship offered by correlation, a simple affine transformation can be used to map to HEP-IR (EGP*FPI) values using Index 5 (eq. 4).

Limitations of this study include the relatively small number of participants investigated, although the number was considerable for a single center cohort with the advantage of using homogenous methods and measurement techniques which reduced variance. Another limitation was that only overweight and obese Caucasian subjects were investigated and therefore results cannot be extrapolated to other groups and ethnicities. Strengths of this study include the detailed phenotypic characterisation of the participants using of state of the art methodology, and the performance of validation studies in a semi-independent cohort. The natural progression for this work is to improve predictability of estimated HEP-IR by incorporating non-linear dynamics in the models, with non-linearity being based on clinical evidence, as opposed to non-causal relationships that may improve regression statistics. An alternative approach is to develop process-driven mathematical models that are derived from descriptions of the physiology, as opposed to the statistical models presented in this paper.

In conclusion, the indices presented in this paper, including that suggested previously [2], have been rigorously tested and validated against a semi-independent cohort. If an index were to be truly predictive it should perform favorably when used against such a data set, yet all the indices shown can only be considered to offer an indication of underlying relationship between the index and HEP-IR, with only marginal benefits over the standard HOMA-IR index. It would therefore be expected that all indices would perform at best with the same level of prediction in a completely independent cohort. However, with reference to Figures 1 and 2, it should be high-lighted that the indices do correlate well with the HEP-IR outcome variable, given the natural variation inherent in biomedical measurements. As such, if a study is willing to accept the approximation such indices offer they may be of use to large epidemiological studies that do not have the facilities or resource to perform stable isotope experiments.

## References

[1] Defronzo RA (2009). Banting Lecture. From the triumvirate to the ominous octet: a new paradigm for the treatment of type 2 diabetes mellitus.. Diabetes.

[2] Vangipurapu J, Stancakova A, Kuulasmaa T, Paananen J, Kuusisto J (2011). A novel surrogate index for hepatic insulin resistance.. Diabetologia.

[3] Weyer C, Bogardus C, Pratley RE (1999). Metabolic characteristics of individuals with impaired fasting glucose and/or impaired glucose tolerance.. Diabetes.

[4] Mari A (2006). Methods of Assessment of Insulin Sensitivity and b-Cell Function.. Immun, Endoc & Metab Agents - Med Chem.

[5] Miyazaki Y, Glass L, Triplitt C, Wajcberg E, Mandarino LJ (2002). Abdominal fat distribution and peripheral and hepatic insulin resistance in type 2 diabetes mellitus.. Am J Physiol Endocrinol Metab.

[6] Hills SA, Balkau B, Coppack SW, Dekker JM, Mari A (2004). The EGIR-RISC STUDY (The European group for the study of insulin resistance: relationship between insulin sensitivity and cardiovascular disease risk): I. Methodology and objectives.. Diabetologia.

[7] Weickert MO, Roden M, Isken F, Hoffmann D, Nowotny P (2011). Effects of supplemented isoenergetic diets differing in cereal fiber and protein content on insulin sensitivity in overweight humans.. Am J Clin Nutr.

[8] Alberti KG, Zimmet P, Shaw J (2005). The metabolic syndrome–a new worldwide definition.. Lancet.

[9] Forster MR (2000). Key Concepts in Model Selection: Performance and Generalizability.. J Math Psychol.

[10] Weickert MO, Arafat AM, Blaut M, Alpert C, Becker N (2011). Changes in dominant groups of the gut microbiota do not explain cereal-fiber induced improvement of whole-body insulin sensitivity.. Nutr Metab (Lond).

[11] Weickert MO, Loeffelholz CV, Roden M, Chandramouli V, Brehm A (2007). A Thr94Ala mutation in human liver fatty acid-binding protein contributes to reduced hepatic glycogenolysis and blunted elevation of plasma glucose levels in lipid-exposed subjects.. Am J Physiol Endocrinol Metab.

[12] Szendroedi J, Schmid AI, Chmelik M, Toth C, Brehm A (2007). Muscle mitochondrial ATP synthesis and glucose transport/phosphorylation in type 2 diabetes.. PLoS Med.

[13] Machann J, Thamer C, Schnoedt B, Haap M, Haring HU (2005). Standardized assessment of whole body adipose tissue topography by MRI.. J Magn Reson Imaging.

[14] Feldstein AE, Wieckowska A, Lopez AR, Liu YC, Zein NN (2009). Cytokeratin-18 fragment levels as noninvasive biomarkers for nonalcoholic steatohepatitis: a multicenter validation study.. Hepatology.

[15] Wieckowska A, Zein NN, Yerian LM, Lopez AR, McCullough AJ (2006). In vivo assessment of liver cell apoptosis as a novel biomarker of disease severity in nonalcoholic fatty liver disease.. Hepatology.

[16] Yilmaz Y (2009). Systematic review: caspase-cleaved fragments of cytokeratin 18 - the promises and challenges of a biomarker for chronic liver disease.. Aliment Pharmacol Ther.

[17] Campioni M, Toffolo G, Basu R, Rizza RA, Cobelli C (2009). Minimal model assessment of hepatic insulin extraction during an oral test from standard insulin kinetic parameters.. Am J Physiol Endocrinol Metab.

[18] Charlton M, Angulo P, Chalasani N, Merriman R, Viker K (2008). Low circulating levels of dehydroepiandrosterone in histologically advanced nonalcoholic fatty liver disease.. Hepatology.

[19] Vuppalanchi R, Chalasani N (2009). Nonalcoholic fatty liver disease and nonalcoholic steatohepatitis: Selected practical issues in their evaluation and management.. Hepatology.

[20] Matthews DR, Hosker JP, Rudenski AS, Naylor BA, Treacher DF (1985). Homeostasis model assessment: insulin resistance and beta-cell function from fasting plasma glucose and insulin concentrations in man.. Diabetologia.

[21] Abdul-Ghani MA, Matsuda M, Balas B, DeFronzo RA (2007). Muscle and liver insulin resistance indexes derived from the oral glucose tolerance test.. Diabetes Care.

[22] Stefan N, Stumvoll M, Vozarova B, Weyer C, Funahashi T (2003). Plasma adiponectin and endogenous glucose production in humans.. Diabetes Care.

[23] Klöting N, Fasshauer M, Dietrich A, Kovacs P, Schon MR (2010). Insulin-sensitive obesity.. Am J Physiol Endocrinol Metab.

[24] Gibbons G (2005). Old fat, make way for new fat.. Nat Med.

[25] Weickert MO, Pfeiffer AF (2006). Signalling mechanisms linking hepatic glucose and lipid metabolism.. Diabetologia.

[26] Rigalleau V, Blanchetier V, Combe C, Guillot C, Deleris G (1997). A low-protein diet improves insulin sensitivity of endogenous glucose production in predialytic uremic patients.. Am J Clin Nutr.

[27] Eidemak I, Feldt-Rasmussen B, Kanstrup IL, Nielsen SL, Schmitz O (1995). Insulin resistance and hyperinsulinaemia in mild to moderate progressive chronic renal failure and its association with aerobic work capacity.. Diabetologia.

